# Effects of External Load and Holding Duration on PAPE and Muscle Activation During Isometric Split Squat Conditioning Activity

**DOI:** 10.3390/medicina62061007

**Published:** 2026-05-22

**Authors:** Mingu Kang, Minsang Kim, Yujin Jeong, Sanghee Park

**Affiliations:** 1 The Integrative Movement Science Laboratory, Gachon University, Incheon 21936, Republic of Korea; mingu1015@gachon.ac.kr (M.K.); minsang304@gachon.ac.kr (M.K.); 17rachel@gachon.ac.kr (Y.J.); 2 Department of Exercise Rehabilitation, Gachon University, Incheon 21936, Republic of Korea

**Keywords:** PAPE, HIMA, split squat, conditioning activity, electromyography, functional movement, vertical jump, kettlebell

## Abstract

*Background* *and Objectives*: Conditioning activities (CAs) are commonly used to induce post-activation performance enhancement (PAPE); however, it remains unclear whether load-dependent responses established in bilateral, predominantly isotonic models extend to unilateral split squat conditions. In particular, evidence regarding holding isometric muscle action (HIMA) is limited, and it is unknown how external load and holding duration interact to influence both performance outcomes and phase-specific muscle activation. Therefore, this study examined the acute effects of HIMA duration and external load during unilateral split squat CA on jump performance and phase-specific electromyographic (EMG) activity. *Materials and Methods*: Twenty recreationally active men completed a randomized, counterbalanced crossover design involving four split squat CA conditions, unloaded 3 s, unloaded 5 s, 3 s loaded (60% 1RM), and 5 s loaded (60% 1RM), each performed as three sets of three repetitions. To minimize fatigue effects, standardized rest intervals and familiarization sessions were implemented prior to testing. Single-leg jump (SLJ) and countermovement jump (CMJ) were assessed before and after CA, with post-activation measurements conducted at 3 min (SLJ) and 4 min (CMJ), consistent with established PAPE time windows. Surface EMG was time-normalized across the split squat cycle and analyzed using phase-specific area under the curve. *Results*: CMJ significantly increased following both loaded conditions (*p* < 0.05; moderate to large effect sizes), whereas no differences were observed between unloaded durations. External load consistently elevated EMG amplitude across all measured muscles (moderate to large effects). Extending duration under load further increased activation in the hamstrings, stabilizers, vastus medialis, and gastrocnemius medialis (*p* < 0.05; small to moderate effects), whereas unloaded conditions showed minimal neuromuscular differences. *Conclusions*: External load, rather than isometric holding duration, appears to be a key factor influencing acute PAPE responses in unilateral split squat HIMA, whereas prolonged holding duration may primarily modulate muscle recruitment patterns without additional performance gains. However, given the acute experimental design and a recreationally active sample, these findings should be interpreted with caution and considered exploratory. Further studies are warranted to confirm these effects across different populations and longer-term training conditions.

## 1. Introduction

Explosive power, defined as the ability to rapidly generate force, is a critical determinant of performance in many sports [1,2]. Accordingly, both long-term training strategies and acute warm-up interventions have been developed to enhance subsequent power-related tasks such as jumping, sprinting, and rapid directional changes [3,4]. Among these, conditioning activities (CAs) are widely implemented, where post-activation performance enhancement (PAPE) refers to a transient improvement in neuromuscular performance following a preceding bout of exercise, including both isotonic and isometric contractions [5,6,7]. The mechanisms underlying PAPE are multifactorial. Acute enhancements in muscle activation, muscle-tendon stiffness, and motor unit recruitment have been proposed as key contributors [5]. In addition, potentiation-related processes such as phosphorylation of myosin regulatory light chains may increase the sensitivity of actin–myosin interactions, thereby facilitating force production and rate of force development [3]. These physiological responses are often indirectly reflected in electromyographic activity, providing insight into neuromuscular readiness following CA.

Most PAPE protocols have traditionally focused on bilateral squat-based exercises. However, many sport-specific movements are performed under unilateral conditions, particularly in sports such as soccer, basketball, track and field, where balance, coordination and limb-specific force production are essential. Previous studies have shown that unilateral strength and power are strongly associated with sprint acceleration, change in direction, and single-leg jump tasks, supporting the relevance of unilateral training approaches in sport-specific contexts [8,9,10]. Consequently, unilateral exercises such as the split squat and Bulgarian split squat (BSS) are increasingly utilized to reflect sport-specific demands and improve functional performance. Despite this relevance, it remains unclear whether the mechanisms underpinning PAPE in bilateral models translate effectively to unilateral tasks, particularly when postural control demands are increased. For example, Papla et al. reported no significant differences in jump or change in direction performance following both bilateral and unilateral complex CA protocols [11]. These findings suggest that biomechanical similarity between CA and performance tasks alone may not be sufficient to induce measurable PAPE. Rather, the effectiveness of PAPE appears to depend on how the conditioning stimulus is structured, including loading intensity, contraction mode, recovery interval, and participant training status [12,13,14]. Therefore, inconsistent findings in the literature may reflect differences in stimulus prescription rather than conflicting underlying mechanisms. Thus, a more controlled examination of these key programming variables within a unified framework is required.

In this context, external load has been a primary variable of interest in optimizing CA protocols. Previous studies using back squat models suggest that moderate to high intensities combined with appropriate recovery intervals (typically 2–12 min) can effectively induce PAPE [12,13,15]. Mechanistically, higher loading intensities are thought to enhance neural drive, increase motor unit recruitment, and elevate muscle-tendon stiffness, all of which may contribute to improved force production and rate of force development [5]. These mechanisms may be particularly relevant in unilateral tasks, where increased load not only amplifies neuromuscular demand but also challenges balance and limb-specific force control. However, whether these dose–response relationships translate to unilateral exercises such as split squats remains unclear. In addition, when isometric muscle actions are incorporated, the influence of external load may differ from that observed in dynamic bilateral models.

Beyond load, isometric contraction strategies have gained attention as a method to enhance neuromuscular activation, particularly in contexts where joint stability and controlled force production are required. Holding isometric muscle actions (HIMAs) can be operationally defined as a sustained isometric contraction performed for a predetermined duration under controlled external load or bodyweight conditions. Unlike maximal voluntary isometric contractions (MVICs), which are typically brief and maximal efforts, HIMA involves submaximal, time-controlled force production that allows for the manipulation of contraction duration as a programming variable. HIMAs using external resistance or bodyweight have been proposed as effective CA modalities [16,17,18,19]. However, the neuromuscular response to isometric exercise appears to depend strongly on how the contraction is implemented, including factors such as contraction duration and external load [16,18,19,20,21,22]. Moreover, findings regarding isometric CA and performance outcomes remain inconsistent. While some studies report enhanced jump performance following isometric conditioning [23], others demonstrated reductions or no meaningful changes in power output [24]. These discrepancies suggest that the effectiveness of isometric CA may depend on specific variables such as contraction duration, external load, and movement configuration. Although both load and duration have been identified as key modulators of neuromuscular responses, it remains unclear how these variables interact within unilateral isometric conditioning tasks and whether their combined effects translate into meaningful performance enhancement.

Accordingly, the purpose of the present study was to investigate the acute effects of two independent variables, which are external load (unloaded vs. loaded) and isometric holding duration (3 s vs. 5 s), on jump performance (SLJ and CMJ) and phase-specific EMG responses during a split squat conditioning activity. Based on the aforementioned gaps, we hypothesized that (1) external load would be the primary determinant of performance enhancement, and (2) holding duration would preferentially influence neuromuscular activation patterns without substantially improving performance outcomes.

## 2. Materials and Methods

### 2.1. Participants

A total of twenty-five recreationally active men were initially recruited. Inclusion criteria required participants to be recreationally active (engaging in structured physical activity ≥2–3 times per week for at least 6 months) and capable of performing a split squat with proper technique. Exclusion criteria included neurological disorders, current or recent (within 6 months) lower limb or lumbar musculoskeletal injury requiring medical treatment, and uncontrolled cardiovascular conditions. Following screening based on predefined eligibility criteria, three individuals were excluded prior to participation due to medical history related to cardiovascular or musculoskeletal concerns, and two withdrew prior to completing the experimental protocol due to personal scheduling conflicts or minor illness. Because these exclusions and withdrawals occurred before the completion of outcome data collection and were not related to the conditioning activity or study outcomes, attrition was considered unlikely to introduce systematic bias. Consequently, twenty participants were included in the final analysis (age: 24.2 ± 5.9 years; height: 173.9 ± 0.9 cm; body mass: 76.6 ± 8.2 kg). The participant recruitment, exclusion, withdrawal, and final inclusion process is summarized in Supplementary Figure S1. All participants were informed of the study procedures both verbally and in writing, and written informed consent was obtained prior to participation. The study protocol was approved by the Institutional Review Board of Gachon University (IRB No. 1044396-202502-HR-039-01, 15 April 2025). Data collection was conducted between September 2025 and February 2026 following a preparatory period after ethical approval. The study was retrospectively registered at the Clinical Research Information Service (CRIS; registration number: KCT0011880). The delayed registration resulted from an administrative oversight, as the registration was initially assumed to be completed but could not be verified in the public registry. Upon identification of this issue, the study was promptly registered. The retrospective registration did not affect the predefined study procedures, outcome measures, data analysis, or reporting of results. All procedures adhered to the Declaration of Helsinki.

### 2.2. Experimental Design

A randomized crossover design with counterbalanced ordering (Latin square) was employed to evaluate PAPE responses across four HIMA split squat conditions: (1) 3 s hold, unloaded (3 s); (2) 5 s hold, unloaded (5 s); (3) 3 s hold with external load at 60% of split squat 1RM (3 s loaded); and (4) 5 s hold with external load at 60% of split squat 1RM (5 s loaded). Participants completed all conditions within a single experimental session following four familiarization visits. To minimize potential fatigue and carryover effects, the order of conditions was counterbalanced, and sufficient rest intervals were provided between conditions, consistent with established acute PAPE protocols. Each CA consisted of three sets of three repetitions, separated by 1 min rest intervals. Given the short duration and low volume of each set (~15–20 s total contraction time), a 1 min rest interval was considered sufficient to minimize fatigue while preserving potentiation effects, consistent with commonly applied PAPE protocols [25]. Prior to testing, a standardized warm-up was performed, followed by baseline measurements of SLJ and CMJ. Three maximal trials were collected for each test (15 s rest between trials; SLJ followed by CMJ). After a 4 min passive recovery period, participants performed condition-specific preparatory sets at low to moderate intensity to standardize movement exposure and minimize unintended variability. The preparatory protocol included unloaded repetitions, submaximal kettlebell loading, and low load HIMA practice at both time durations. All preparatory activities were standardized across participants in terms of sequence, intensity, and supervision to ensure consistent movement exposure and minimize variability prior to the conditioning activity. Subsequently, participants performed the assigned CA condition. Based on pilot testing, loads exceeding approximately 70% 1RM frequently resulted in technical failure (e.g., loss of balance or kettlebell instability). Therefore, 60% of split squat 1RM was selected as the highest tolerable intensity that could be consistently executed with proper technique across participants, supporting its selection as the highest feasible and reliable loading condition. This loading range is also consistent with previous PAPE studies demonstrating that moderate to high intensities are effective for inducing potentiation while maintaining movement quality [13,25]. Post-CA performance tests were conducted at 3 min (SLJ) and 4 min (CMJ), aligning with established PAPE time windows [3,26]. This recovery interval was selected based on previous evidence indicating that PAPE responses typically occur within a 2–12 min window following moderate-to-high intensity conditioning activities, with the most pronounced effects commonly observed around 3-5 min [3,26]. Although the optimal recovery time may vary between individuals, fixed time points (3–4 min) were used to standardize post-activation measurements and allow consistent within-subject comparisons across conditions. This procedure was repeated for all four conditions.

### 2.3. Warm-Up and Familiarization

All sessions incorporated a standardized general warm-up consisting of dynamic mobility and activation exercises (leg swings, lunges, squats, and whole-body movements). During familiarization, participants practiced all HIMA conditions under investigator supervision. Isometric holding durations were controlled using a stopwatch, initiated when the lead leg reached approximately 90° knee flexion and terminated upon a verbal cue from the investigators. Each duration (3 s and 5 s) was repeated multiple times to ensure consistency. Load familiarization was progressed across visits. Initial sessions used estimated loads derived from self-reported back squat 1RM (approximately 75% conversion to split squat 1RM [27]); kettlebell loads equivalent to around 37.5% of self-reported back squat 1RM were used for load familiarization in HIMA conditions. The self-reported 1RM was used solely to estimate initial submaximal loads during first and second familiarization. Later sessions (familiarization visits 3–4 after the split squat 1RM assessment) employed 60% of directly assessed split squat 1RM. Jump testing procedures were also practiced to minimize potential learning effects. The preparatory protocol was standardized across all sessions and designed to ensure movement consistency and baseline readiness without inducing fatigue or additional potentiation. This approach minimized variability while maintaining ecological validity for performance testing. All timing procedures were administered by the same investigator, and participants practiced each condition repeatedly during familiarization to ensure consistent execution. This approach has been widely adopted in time-controlled isometric protocols [25,28] and provides sufficient reliability for short-duration contractions.

### 2.4. Split Squat 1RM Assessment

Split squat 1RM was estimated using a progressive submaximal protocol. The submaximal estimation approach has been widely used in strength assessment and is considered appropriate for unilateral tasks where direct 1RM testing may be limited by balance and technical constraints. Due to the technical demands of unilateral loading, an 8–11 repetition maximum estimation approach was adopted rather than direct maximal testing. Participants performed up to three trials with incremental load adjustments. Initial trials targeted approximately 11RM, followed by refinement based on performance, and a final trial approximating 8RM to ensure estimation accuracy. Trials were considered invalid if movement cadence deviated or balance was compromised. Verbal encouragement was provided to promote maximal effort.

### 2.5. Anthropometrics and Movement Standardization

Anthropometric measures (height, body mass, blood pressure, leg length, and limb dominance) were recorded during the initial visit. Leg length was defined as the distance from the anterior superior iliac spine to the medial malleolus. Split squat positioning was standardized relative to individual anthropometrics: the step length was set to 100% (±5%) of the leg length, and the step width to 75% (±5%) of the hip width [29]. During all trials, the medial arch of the dominant foot was positioned at the center of the force plate. Rear foot placement was standardized according to the predetermined leg length and width with the hallux aligned to a pre-marked reference point to ensure consistent positioning across sessions. All measurements and positioning procedures were performed by the same investigator, and pre-marked reference points were used to ensure consistent placement across sessions.

### 2.6. Movement Execution and Behavioral Control

All repetitions were performed under controlled range of motion. Participants descended until the lead thigh was approximately parallel to the ground, maintained an isometric muscle action for 3 s or 5 s and then returned to full extension without losing balance. Visual fixation was standardized by instructing participants to focus on a target positioned at eye level. In addition, participants were instructed to maintain consistent lifestyle behaviors and to refrain from strenuous exercise, caffeine, and alcohol for at least 48 h prior to testing. Compliance with these instructions was confirmed verbally prior to each testing session. All testing sessions were conducted at a similar time of day for each participant.

### 2.7. Outcome Measures

#### 2.7.1. Jumping Performance

SLJ and CMJ were assessed using a force platform (BMS600600-2000, Watertown, MA, USA), sampling at 1000 Hz [30]. The force platform was zeroed prior to each testing session following the manufacturer’s standard procedure to ensure baseline accuracy. The validity and reliability of force platforms for jump assessment have been well established in the previous literature [31]. Jump height was calculated from the flight time (FT), defined as the time interval between take-off and landing. Based on projectile motion principles, jump height (h) was computed as follows:

$$Jump\;height=\frac{\left(g\times FT^{2}\right)}{8}$$

where *g* represents the acceleration of gravity (9.81 m/s^2^) [32]. Take-off and landing were identified using a threshold of 20 N in vertical ground reaction force (vGRF), with take-off defined as the point when vGRF fell below 20 N and landing when it exceeded 20 N [33]. For SLJ, arm movement was restricted, and the non-dominant leg was stabilized to eliminate confounding effects [9]. Upon a verbal “ready” signal from the investigator, participants stepped on a force platform and on the signal “go”; participants performed a maximal vertical jump and were instructed to land at approximately the same location on the force platform. CMJ was performed bilaterally with self-selected depth [34]. Three maximal trials were collected per condition.

#### 2.7.2. Surface Electromyography

Muscle activity was recorded using wireless surface electromyography (sEMG) (TELEmyo DTS; Noraxon USA Inc., Scottsdale, AZ, USA) at 2000 Hz. Signals were band-pass filtered (20–500 Hz; fourth-order Butterworth), rectified, and smoothed using root mean square (RMS) (100 ms window) in Noraxon Myo-Research XP Master Edition software (Noraxon USA, Inc.). All MVIC procedures were standardized and performed under consistent conditions to minimize variability across sessions. RMS amplitudes were then normalized to each participant’s maximal voluntary isometric contraction (MVIC) and expressed as a percentage of MVIC (%MVIC). Electrodes (Bio-Protech Inc., Tustin, CA, USA) (diameter: 10 mm, bipolar configuration, and interelectrode distance > 20 mm) were used [35]. The surface electrodes were placed on the gluteus medius (Gmed), biceps femoris (BF), semitendinosus (ST), vastus medialis (VM), vastus lateralis (VL), peroneus longus (PL), gastrocnemius medialis (GM), and gastrocnemius lateralis (GL). Prior to electrode placement, general warm-up was completed and each participant’s skin was shaved and cleansed with alcohol to reduce impedance. Electrodes were positioned over the muscle belly, aligned with the direction of muscle fibers, according to SENIAM recommendations. Placement sites were identified based on standardized anatomical landmarks and marked to ensure consistent positioning across sessions, and leads were secured using elastic adhesive tape to minimize movement artifacts.

#### 2.7.3. MVIC Normalization

Maximum voluntary isometric contractions were collected for each muscle using standardized testing positions (e.g., hip abduction for Gmed, knee flexion for BF and ST, knee extension for VM and VL, ankle plantarflexion for GM and GL, and ankle eversion for PL) following established protocols [36,37]. Three trials (5 s each) were performed with sufficient rest between trials. The highest RMS value was used for normalization and EMG amplitudes were expressed as %MVIC. Verbal encouragement was provided to ensure maximal effort.

### 2.8. Statistical Analyses

Sample size estimation was conducted using G*Power (v3.1; Universität Kiel, Kiel, Germany), assuming a medium effect size of 0.5, alpha = 0.05, and power = 0.80, resulting in a required sample size of 20 participants (*n* = 20). The final sample size met the a priori power requirement for the repeated-measures design and is consistent with previous acute PAPE studies employing within-subject experimental frameworks [26,38,39]. The chosen medium effect size (Cohen’s d = 0.5) was verified by prior performance and EMG studies which demonstrated noticeable magnitudes of changes in jump performance and EMG activation following similar interventions [3,28,37]. In addition, the repeated-measures crossover design increases statistical power by reducing inter-individual variability, supporting the adequacy of the calculated sample size. Normality of the dependent variables was assessed using the Shapiro–Wilk test prior to inferential analysis. For repeated-measures factors, sphericity was evaluated using Mauchly’s test, and Greenhouse–Geisser correction was applied when the assumption of sphericity was violated. For statistical comparison, a two-way repeated measures ANOVA followed by Holm–Sidak multiple comparisons test was applied to assess differences in SLJ, CMJ, and EMG outcomes across CA conditions on the main effect of time duration and external load, as well as their interaction. Data were presented as the mean ± standard error of the mean (SEM) to indicate the precision of the estimated condition mean, with individual participant data points overlaid in the figures to show inter-individual variability. Statistical significance was set at *p* < 0.05. Outliers were identified using a pre-defined criterion (e.g., >2 standard deviation from the participant’s condition mean) and excluded consistently across all conditions. In most cases, this involved the removal of ≤1 data points per condition, with several conditions showing no outliers. Although a small number of conditions exhibited a higher number of outliers (up to two data points per condition), the overall proportion remained low relative to the sample size. Analyses with and without outlier exclusion yielded comparable results. Analyses were performed using GraphPad Prism (version 11.0.0; GraphPad Software, San Diego, CA, USA). Effect sizes were calculated and reported as Cohen’s d for pairwise comparisons and partial eta squared (ηp^2^) for ANOVA effects. All statistical assumptions were checked prior to analysis and appropriate corrections were applied where necessary.

## 3. Results

Outcomes were analyzed in two domains: jump performance (SLJ and CMJ) following each CA and muscle activation patterns during the CA. Jump performance was expressed relative to pre-test values (pre-test = 100%). EMG signals were time-normalized across the split squat cycle and analyzed using the phase-specific area under the curve (AUC) for the descending, HIMA and ascending phases. The dynamic portion of the movement (descending and ascending phases) was normalized to 100% (50% each) while the isometric holding phase (HIMA) was normalized separately to 100%. This resulted in a combined normalized cycle of 200%, allowing direct comparison of neuromuscular activation across dynamic and isometric contraction phases. This approach was adopted to preserve phase-specific characteristics and avoid the loss of information associated with traditional peak or mean EMG analyses. Table 1 summarizes the overall significant effects for jump performance and EMG outcomes.

### 3.1. Jump Performance

**Figure 1:**
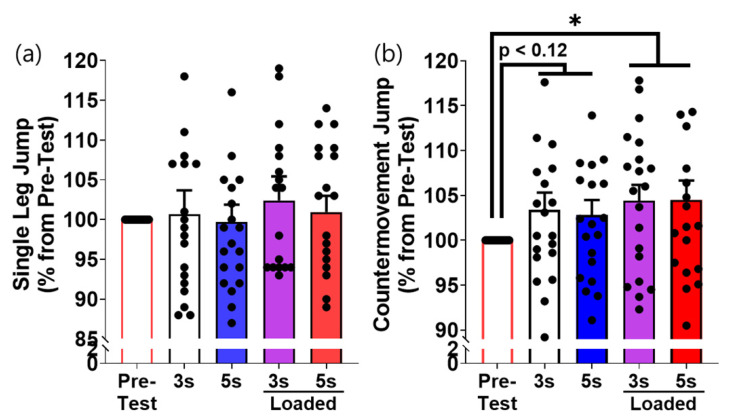
Jump performance following conditioning activities. (**a**) Singl-leg jump and (**b**) countermovement jump, expressed as a percentage change from the pre-test. CMJ showed the main effects of time duration and external load. Bars indicate mean ± SEM to show the precision of the estimated condition mean while individual dots represent participant-level values and data dispersion. * indicates statistical significance between the indicated comparison.

CMJ responses differed depending on external load, whereas SLJ remained unchanged across all conditions (Figure 1). In unloaded conditions, CMJ values were slightly higher than pre-test; however, these differences were not statistically significant (3 s: *p* = 0.09; 5 s: *p* = 0.12). In contrast, both loaded conditions (3 s loaded and 5 s loaded) significantly increased CMJ performance relative to the pre-test (*p* < 0.05; Figure 1b) with mean increases of approximately 4.3% and 4.4%, respectively. In addition, the mixed-effects analysis revealed a significant main effect of load, with loaded conditions showing a higher predicted CMJ value than unloaded conditions by 1.30% (95% CI: 0.0004 to 2.61; Cohen’s d = 0.47).

### 3.2. Muscle Activation

**Figure 2:**
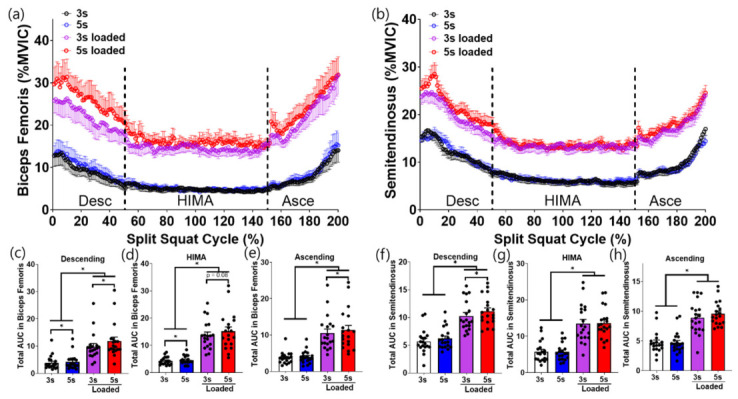
Hamstring muscle activation during conditioning activities. (**a**,**b**) Averaged EMG profiles of biceps femoris (BF) and semitendinosus (ST) across the split squat cycle. (**c**–**h**) Phase-specific AUC for descending, HIMA and ascending phases. A time duration effect was observed in the descending phase, and a load and duration interaction was identified in BF. Bars indicate the mean ± SEM to show the precision of the estimated condition mean while individual dots represent participant-level values and data dispersion. * indicates statistical significance between the indicated comparison. AUC: area under the curve.

**Figure 3:**
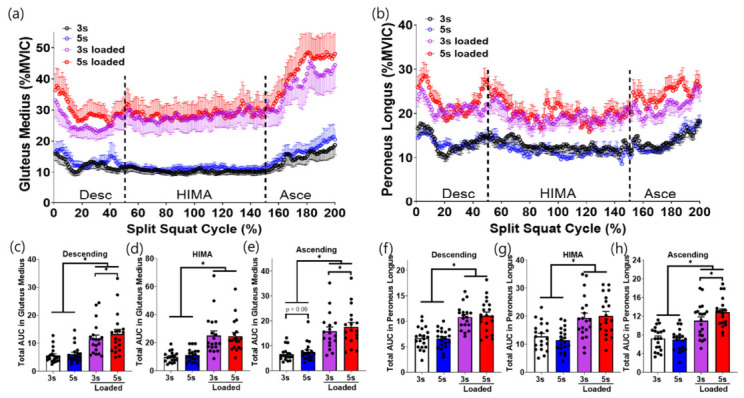
Stabilizer muscle activation during conditioning activities. (**a**,**b**) Averaged EMG profiles of the gluteus medius (GM) and peroneus longus (PL) across the split squat cycle. (**c**–**h**) Phase-specific AUC for descending, HIMA and ascending phases. A time duration effect was observed during the ascending phase for both GM and PL, and a significant interaction effect was identified in the ascending phase for PL. Bars indicate the mean ± SEM to show the precision of the estimated condition mean while individual dots represent participant-level values and data dispersion. * indicates statistical significance between the indicated comparison. AUC: area under the curve.

**Figure 4:**
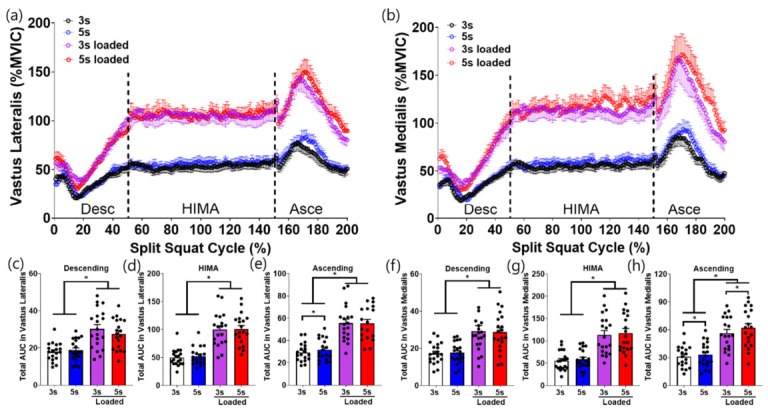
Quadricep muscle activation during conditioning activities. (**a**,**b**) Averaged EMG profiles of vastus lateralis (VL) and vastus medialis (VM) across the split squat cycle. (**c**–**h**) Phase-specific AUC for descending, HIMA and ascending phases. Time duration effects were observed during the ascending phase in both VL and VM and a significant interaction effect was identified in the ascending phase for VM. Bars indicate the mean ± SEM to show the precision of the estimated condition mean while individual dots represent participant-level values and data dispersion. * indicates statistical significance between the indicated comparison. AUC: area under the curve.

**Figure 5:**
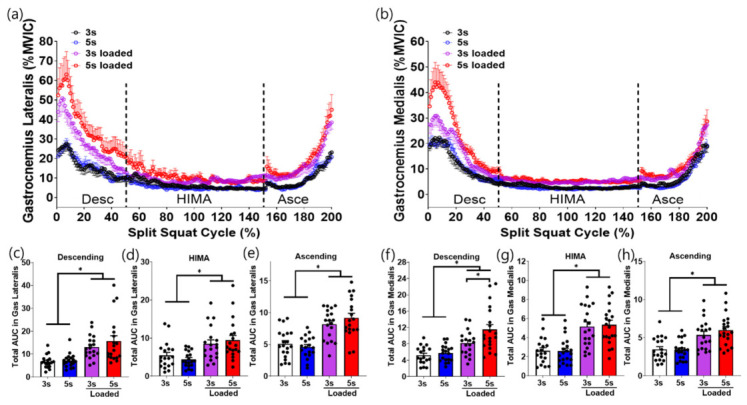
Gastrocnemius muscle activation during conditioning activities. (**a**,**b**) Averaged EMG profiles of gastrocnemius lateralis (GL) and gastrocnemius medialis (GM) across the split squat cycle. (**c**–**h**) Phase-specific AUC for descending, HIMA and ascending phase. A time duration effect was observed in the descending phase of GM with an interaction effect identified in the ascending phase of GL. Bars indicate the mean ± SEM to show the precision of the estimated condition mean while individual dots represent participant-level values and data dispersion. * indicates statistical significance between the indicated comparison. AUC: area under the curve.

Overall, external load exerted a consistent main effect across all phases and muscle groups, whereas time duration and interaction effects were muscle- and phase-specific (Figure 2, Figure 3, Figure 4 and Figure 5).

As for the external load response (loaded vs. unloaded), across all measured muscles, load conditions (3 s loaded and 5 s loaded) significantly increased EMG amplitude compared with unloaded conditions (*p* < 0.05; Cohen’s d = 0.6–1.2). The effect was observed in the hamstrings (biceps femoris, semitendinosus), stabilizing muscles (gluteus medius, peroneus longus), quadriceps (vastus lateralis, vastus medialis), and plantar flexors (gastrocnemius), with corresponding increases in the phase-specific AUC during the descending, HIMA and ascending phases.

As for the time duration response (3 s vs. 5 s, unloaded), extending the holding duration from 3 s to 5 s produced selective, rather than uniform, increases in EMG activation. Significant duration effects were observed mainly in the biceps femoris during the descending and HIMA phases (*p* < 0.05; Figure 2c,d), and quadriceps during the ascending phase (*p* < 0.05; Figure 4e,h). Additionally, the gluteus medius showed a trend toward increased activation during the ascending phase (*p* = 0.09; Figure 3e).

As for the interaction response (load and duration), significant load x duration interactions were observed in several muscles and phases (*p* < 0.05, ηp^2^ = 0.15–0.32). Under loaded conditions, extending the holding duration (5 s loaded vs. 3 s loaded) resulted in greater muscle activation in specific muscles and phases. During the descending phase, 5 s loading elicited significantly higher activation in the biceps femoris, semitendinosus, gluteus medius and gastrocnemius medialis (*p* < 0.05; Figure 2c,f, Figure 3c and Figure 5f), with moderate to large effect sizes. During the ascending phase, 5 s loading further increased activation in the biceps femoris, gluteus medius, peroneus longus and vastus medialis (*p* < 0.05; Figure 2e, Figure 3e,h and Figure 4e).

## 4. Discussion

The present study demonstrated that external loading during a split squat conditioning activity produced significant improvements in CMJ performance, whereas extending isometric holding duration alone did not provide additional jump performance benefits. From a physiological perspective, the superior effect of loading may be explained by greater neural drive and motor unit recruitment, as well as increased muscle-tendon stiffness, which are known contributors to PAPE. EMG findings further showed that loaded conditions elicited a global increase in lower-limb muscle activation while duration-dependent effects were more selective and muscle-specific. These results indicated that external loading may have contributed to performance-relevant potentiation by increasing overall neuromuscular demand, whereas holding duration appeared to modulate recruitment patterns rather than directly enhancing jump output. Accordingly, the study hypothesis was partially supported: external loading enhanced CMJ performance and broadly increased lower-limb muscle activation, whereas prolonged isometric holding duration primarily influenced muscle-specific recruitment patterns without further improving jump performance.

### 4.1. PAPE Response

The observed PAPE in the current study aligns with the established PAPE literature, which shows that the effectiveness of a conditioning activity is determined more by the magnitude of the neuromuscular stimulus, including intensity, contraction mode, recovery interval, and participant training status [7,13,40]. Previous studies have reported inconsistent PAPE outcomes when conditioning activities lacked sufficient loading stimulus, even when the conditioning task was biomechanically similar to the target movement, as shown by Papla et al. [11]. Conversely, moderate-to-high intensity resistance exercises have shown more reliable improvements in explosive performance [12,13,15]. Thus, the absence of potentiation in some studies does not necessarily contradict the present findings, but rather indicates that similar movement patterns may produce different outcomes depending on how the conditioning stimulus is prescribed. Within this context, the current findings extend prior work by showing that an isometric split squat can function as an effective PAPE stimulus when combined with sufficient external load.

### 4.2. Effect of External Loading

A key finding of the current study was that external loading was associated with a broad increase in activation across the lower-limb musculature, including the hamstrings, quadriceps, plantar flexors and stabilizing muscles. This activation pattern may reflect increased neuromuscular demand during the conditioning activity and could be required for the observed improvement in CMJ performance. Explosive tasks such as CMJ require coordinated force generation across the hip–knee–ankle kinetic chain, and loaded conditions may enhance this coordination by simultaneously increasing activation capacity across multiple muscle groups [5,7,13]. Importantly, the selected loading intensity (60% 1RM) represented the highest tolerable level that participants could maintain during isometric holding with minimal technical failure, thereby providing a strong yet feasible conditioning stimulus. However, because direct kinetic or kinematic measurements were not included, these interpretations should be considered inferential. The present EMG findings primarily indicate increased neuromuscular demand rather than directly confirming changes in force production or inter-segmental coordination.

### 4.3. Effect of Isometric Holding Duration

In contrast to the clear effect of loading, prolonging isometric holding duration (5 s vs. 3 s) did not further enhance jump performance, even under loaded conditions. However, duration-dependent changes were evident at the neuromuscular level. Specifically, the 5 s loaded condition elicited greater activation in selected posterior chain muscles, particularly the hamstrings and planter flexors across the descending phase. This dissociation suggests that holding duration may influence muscle recruitment strategy rather than increasing total force output. Previous studies have shown that sustained isometric contractions can increase muscle activation over time, particularly under loading conditions [41]. However, such increases do not necessarily translate to improved explosive performance. Therefore, the present findings suggest that the neuromuscular effects of prolonged isometric contraction are highly dependent on loading context and may reflect adjustments in motor unit recruitment associated with increased time-under-tension. Under unloaded conditions, extending duration had minimal impact on muscle activation, whereas under loaded conditions, extending duration selectively enhanced posterior chain engagement, showing interactive responses in the gluteus medius, biceps femoris, semitendinosus, and gastrocnemius medialis across the descending phase. From a practical standpoint, this implies that duration manipulation alone is insufficient to augment PAPE but may be useful for targeting specific muscle recruitment patterns when combined with sufficient load.

### 4.4. Limitations and Future Research Directions

Several limitations should be considered when interpreting these findings. First, the study examined only acute responses, and therefore does not capture how repeated exposure to isometric conditioning may influence long-term neuromuscular adaptation or performance retention. Future studies should investigate whether the load- and duration-dependent responses observed here translate into chronic training adaptations. Second, the sample consisted of recreationally active young men, which may restrict generalizability to female athletes, older populations, or highly trained individuals. Further research should examine whether gender, age, or training status modifies PAPE responses to loaded isometric conditioning. Third, the present analysis focused on jump performance and surface EMG without direct kinematics or kinetic measurement. Therefore, future studies should incorporate force production, joint kinetics, and movement coordination variables to clarify the mechanisms underlying the observed performance and EMG responses. Fourth, because the experimental protocol was conducted within a single session, potential fatigue or carryover effects cannot be entirely excluded despite standardized rest intervals and randomized crossover ordering. In addition, blinding was not feasible due to the nature of the interventions, which may have introduced expectancy-related bias. Finally, surface EMG is subject to inherent limitations, including its inability to fully capture deep muscle activity, which should be considered when interpreting neuromuscular responses.

### 4.5. Practical Applications

From a practical perspective, external load should be prioritized when designing split squat-based conditioning activities to induce acute PAPE responses. Moderate loading, such as approximately 60% 1RM, may be sufficient to enhance subsequent CMJ performance under field-feasible conditions. In contrast, extending isometric holding duration alone appears unlikely to provide additional jump performance benefits. However, manipulating holding duration under loaded conditions may be useful for selectively targeting neuromuscular recruitment, particularly within the posterior chain. Therefore, practitioners may consider load the primary programming variable for performance enhancement while using holding duration as a secondary variable for muscle-specific activation goals.

## 5. Conclusions

External load during split squat conditioning activity was associated with improved CMJ performance and a broad increase in lower-limb muscle activation in recreationally active young men. In contrast, prolonged isometric holding duration appeared to influence muscle recruitment patterns without providing additional jump performance benefits. Thus, the study hypothesis was partially supported: load prescription appeared to be a more influential factor for eliciting acute PAPE responses while holding duration may serve as a secondary variable for targeting specific neuromuscular responses.

## Figures and Tables

**Table 1:** Summary of significant effects for jump performance and EMG outcomes. ns indicates not significant.

Outcome	Main Finding	Statistical Summary
CMJ	Loaded conditions increased CMJ relative to pre-test	*p* < 0.05
SLJ	No significant change across conditions	ns
External load	Increased EMG across most muscles and phases	*p* < 0.05; Cohen’s d = 0.6–1.2
Time duration	Selective increases in BF, quadriceps, and GM	*p* < 0.05
Load × duration	Significant interactions in selected muscles/phases	*p* < 0.05; ηp^2^ = 0.15 – 0.32

## Data Availability

The original contributions presented in this study are included in the article/Supplementary Materials. Further inquiries can be directed to the corresponding author.

## Supplementary Materials

The following supporting information can be downloaded at https://www.mdpi.com/article/10.3390/medicina62061007/s1, Figure S1: Participant flow diagram. A total of 25 recreationally active men were initially recruited. Three individuals were excluded before participation due to cardiovascular or musculoskeletal medical history, and two withdrew before completing the experimental protocol due to scheduling conflicts or minor illness. Consequently, 20 participants completed the randomized counterbalanced crossover protocol and were included in the final analysis.
