# Supplementary material for: Effects of External Load and Holding Duration on PAPE and Muscle Activation During Isometric Split Squat Conditioning Activity
Source: Medicina (Kaunas). 2026 May 22;62(6):1007. doi: 10.3390/medicina62061007 (PMC13303738; doi:10.3390/medicina62061007)
Supplement: Supplementary file 1 [file medicina-62-01007-s001.zip › medicina-4263992-supplementary.pdf]

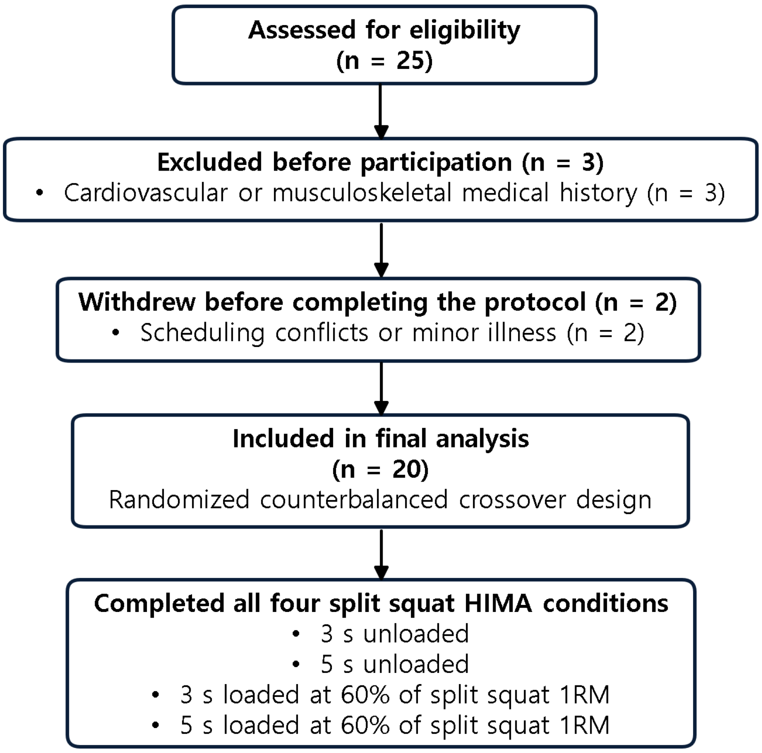

Supplementary Figure S1. Participant flow diagram. A total of 25 recreationally active men were initially recruited. Three individuals were excluded before participation due to cardiovascular or musculoskeletal medical history, and two withdrew before completing the experimental protocol due to scheduling conflicts or minor illness. Consequently, 20 participants completed the randomized counterbalanced crossover protocol and were included in the final analysis.
